# Food Media and Dietary Behavior in a Belgian Adult Sample: How Obtaining Information From Food Media Sources Associates With Dietary Behavior

**DOI:** 10.3389/ijph.2022.1604627

**Published:** 2022-05-23

**Authors:** Viktor Lowie Juliaan Proesmans, Iris Vermeir, Charlotte de Backer, Maggie Geuens

**Affiliations:** ^1^ Department of Marketing Innovation and Organisation, Ghent University, Ghent, Belgium; ^2^ Department of Communication Studies, University of Antwerp, Antwerp, Belgium

**Keywords:** media, nutrition, public health, dietary preferences, influencer, celebrity chef

## Abstract

**Objective:** We aim to relate Flemish adults’ main food information sources (e.g., celebrity chefs, experts) with their dietary behavior.

**Methods:** A cross-sectional online survey among 1115 Flemish adults who regularly cook, measured the food information sources the respondents used to obtain recipes, their dietary intake and dietary restrictions. Ordinal and logistic regression were used to investigate the relation between food media, dietary intake and dietary restrictions.

**Results:** Celebrity chefs were mentioned most often (37%) as main food information source, followed by family and acquaintances (21%) and lifestyle gurus (12%). Using lifestyle gurus as a source of dietary information is associated with more dietary restrictions and a higher intake frequency of plant-based food groups, whereas using celebrity chefs or experts is associated with a different (but less unequivocal vegetarian or healthy) dietary intake.

**Conclusion:** Media icons like lifestyle gurus and celebrity chefs appear to be among people’s main sources of food information. There is a significant association between using them as a source of food information and dietary behavior. Further research on the influence of media on diet is required.

## Introduction

Since the 1970s, western diets increasingly consist of processed foods rich in saturated fat, sugar, and sweeteners; a trend ultimately crossing the globe resulting in an obesity epidemic [1, 2]. Compared to 1975, global obesity rates have almost tripled, with 39% of men and 40% of women being overweight or obese in 2016 [3]. Similarly, according to Eurostat, 53% of the EU population was overweight among which 17% was obese in 2019 [4]. With poor diet and obesity being important risk factors for most non-communicable diseases, obesity manifests a high burden on public health [5, 6]. On a personal level, obese people have a 32% higher medical spending compared to normal weight people [6]. On a societal level, the OECD countries estimate to spend 8.4% of their health budget on treating the consequences of high body mass in the period 2020–2050 [7].

The foregoing numbers indicate obesity prevention is highly called for. Unfortunately, governmental efforts to promote healthier food choices so far seem to have little effect. While people consider health experts and governmental agencies to be trustworthy, adherence to nutritional guidelines remains rather low, with people perceiving the information as confusing and hard to understand [8, 9]. Meanwhile, data from the US, Finland, Canada, Norway and Australia show how mass media, and in particular the internet, has become an increasingly prominent source of food information in Western societies over the past two decades [10, 11].

The increasingly prominent role of mass media as a source of food information started with the boom in cooking television at the start of the twenty-first century [12, 13]. TV chefs achieved a celebrity-like status and multiple countries like the US, UK and Belgium got their own TV network solely broadcasting cooking shows [12, 13]. While the recipes used in these cooking shows and promoted by celebrity chefs have been criticized in the past for being too rich in protein, total fat, saturated fatty acids, and salt, it is unclear whether they have any effect on the dietary behavior of their viewers [14–16]. Some studies suggest that cooking shows and celebrity chef content can increase the intake of specific food groups as well as the time spent on cooking among their viewers [12, 17–19]. Other studies reject this notion, claiming people consume celebrity chef content rather as entertainment, finding hardly any relation with dietary behavior [20, 21].

Besides cooking television, online media has become an increasingly prominent source of food information. A review from Boylan et al. [8] even reported online media as people’s main source of dietary information. Clarke et al. [22] reported how celebrity chefs are widely prominent on multiple social media platforms such as Facebook, Instagram, and Pinterest. Similarly, Elias [23] pointed out that online media has democratized food writings as everybody can share their own recipes with a wider audience. This gave opportunity to lifestyle gurus or influencers, defined by Baker and Rojek [24] as “unlicensed native agents of awareness, positioned in conventional and social media, to offer emotional support, an identity matrix and pedagogy for self-discovery and well-being” to gain prominence. Online media made it easier than ever to establish a forum and promote certain diets or lifestyles, even if the claims made are not scientifically substantiated.

While also lifestyle gurus have received criticism from the scientific community [9, 25], their followers perceive them as authentic and trustworthy due to their personal discourse and the role model function they portray [26–28]. As is the case for celebrity chef content, it is unclear how content provided by lifestyle gurus affects dietary behavior. A survey from Byrne et al. [27] found lifestyle gurus to be highly influential for 16% of the respondents, while 32% of the respondents reported to be motivated to eat healthier. This may, however, be deceptive, as lifestyle gurus advocate for healthy diets by “clean eating” in which nutrients and foods like carbohydrates, dairy, fat or sugar ought to be restricted, something that is not in line with dietary guidelines [27]. Looking at recipes provided by lifestyle gurus on online blogs, they were higher in protein and fat compared to other online recipes, while there was no difference in sugar or salt [29]. This does not insinuate a healthier diet, as the recipes of popular food blogs already contain too high amounts of saturated fat and salt [30].

Considering the increasing influence of mass media and the possible implications it has on dietary behavior, the aim of this study is to examine to what extent the Flemish adult population uses mass media content to obtain food information and how using this information associates with dietary behavior, as measured by dietary intake and dietary restrictions.

## Methods

### Study Design

A cross-sectional online survey was conducted among a sample of the adult Flemish population. The survey was anonymous and could be filled in at any time. Data collection occurred in November and December 2019.

### Sample

Respondents were recruited from the online consumer panel maintained by Dynata, a professional marketing research agency. The requirements for enrollment were being 18 years or older and cooking at least once a month. As there is no data on which percentage of the population in this age group cooks at least once a month the study aimed for a diverse sample in terms of sex, age groups, education, and diet. Of the 1535 respondents who filled in the questionnaire, 420 respondents were excluded because they did not meet the inclusion criteria, did not fill in every question, or because they stopped prematurely. Therefore, 1115 respondents were retained for analysis. Table 1 shows the baseline characteristics of the included respondents.

**TABLE 1:** Sample characteristics (*n* = 1115) (association between media and diet, Belgium, 2022).

Sex	Male (%)	51.0	First Nationality, Belgian (%)	96.5
	Female (%)	49.0	BMI, mean (SD)	25.9 (4.96)
Age group	18–34 (%)	24.1
	35–49 (%)	20.6	Following a diet (%)	8.1
	50–64 (%)	24.4	Flexitarian^a^ (%)	8.3
	65+(%)	31.0	Vegetarian^b^ (%)	2.7
BMI group	Underweight BMI< 18.5 kg/m^2^ (%)	2.8	Pescatarian^c^ (%)	2.4
	Healthy weight (BMI ≥18.5 kg/m^2^, and <25 kg/m^2^) (%)	40.3	Vegan^d^ (%)	2.5
	Overweight (BMI ≥25 kg/m^2^, and <30 kg/m^2^) (%)	37.0	Suffers from a food intolerance (%)	5.0
	Obese (BMI ≥30 kg/m^2^) (%)	19.9	Suffers from a food allergy (%)	5.0
Education	Lower education (%)	21.3	Has religious restrictions (%)	2.4
	Middle education (%)	39.1		
	Higher education (%)	39.6		

a Consciously avoiding meat at least a few days a week.

b Consciously avoiding meat and fish.

c Consciously avoiding meat, while still consuming fish.

d Consciously avoiding all foods derived from animals.

Due to a lack of the population characteristics that regularly cooks, we compare the study sample to the full adult Flemish population. This comparison shows that although the study sample is not representative for the Flemish population, it is very diverse. Specifically, according to the national health survey (2018), the average Body Mass Index (BMI) in Flanders is 25.3 kg/m^2^, compared to 25.9 kg/m^2^ in the study sample (t = 3.932, *p* < 0.001) [31]. Furthermore, 2.9% of the Flemish population is underweight, while 48.2% is overweight and 15.0% is obese. In the study sample these percentages amount to 2.8%, 37.0% and 19.9% respectively. As such, the study sample had more obese but fewer overweight people compared to the general population (χ^2^ = 60.38, *p* < 0.001) [31]. The study sample also slightly (but significantly) differed from the general Flemish population in terms of education level: 39.6% of the study sample had a higher education, 39.1% had a middle education, and 21.3% had a lower education. Among the Flemish population this is 41%, 41%, and 18% respectively (χ^2^ = 8.26, *p* = 0.016) [32]. Finally, the study sample was significantly older than the national Flemish adult population, with 24.1% of the respondents being in the 18–34 age group, 20.6% being in the 35–49 age group, 24.4% being in the 49–64 age group, and 31% being in the 65+ age group, as compared to 24.9%, 24.1%, 25.9%, and 25.1% in the population, respectively (χ^2^ = 20.55, *p* < 0.001) [32].

### Survey

The survey was developed using the online survey software Qualtrics. Before participation, respondents were asked for their informed consent and were informed that they could terminate the participation at any point.

The survey consisted of three parts. First, respondents were asked who/what were the most influential sources for food information and recipes for them. This was an open-ended question, and multiple sources could be reported. The second part of the survey was a validated short food frequency questionnaire (SFFQ) to measure the intake frequency of different food groups [33]. While the original SFFQ comprised 19 items regarding different food groups (e.g., fruit, vegetables), another 11 items were added as the original questionnaire did not include food groups like nuts and seeds or legumes which are widely consumed and discussed in dietary guidelines (Supplementary Appendix S1) [34]. Respondents had to fill in how many times a week they generally consume food products from each category. This could either be “never,” “less than once a week,” “once a week,” “2–4 days a week,” “5–6 days a week,” “once a day,” or “more than once a day.” The survey did not require participants to indicate portion sizes. The final part of the questionnaire comprised questions regarding dietary restrictions, BMI, diet, and sample characteristics. Dietary restrictions were measured by asking the respondents on a dichotomous scale (yes or no) whether they are on a diet, generally avoid certain food components or specifically avoid fat, sugar, dairy, or carbohydrates. These food components were chosen as they seem to be restricted in many popular fad diets [35].

For the sample characteristics, respondents were asked to record their weight (in kg) and height (in cm), whether they had any intolerances, allergies, or religious restrictions (yes or no) and to select their sex (male/female/other), age (18–100), first nationality, and highest obtained educational degree. If people did not obtain a high school degree, they were labeled as having a lower education. If people had a high school degree but no Bachelor’s degree, they were labeled as middle educated; if people had at least a Bachelor’s degree they were labeled as having a higher education.

### Coding and Statistical Analysis

After analyzing respondents’ answers to the question which food information sources they use to obtain recipes and food information, nine different groups of food information sources were identified. Based on these nine different groups, a coding scheme (Table 2) was developed to code all responses. When it was unclear in which category a given response should be classified (e.g., cookameal was classified as online media after verification), the online search engine Google was used for clarification. Furthermore, an 10th category of “others” was used for answers that did not fit any of the other categories and were too small to form its own category (e.g., meal boxes like Hello Fresh). As respondents could use multiple food information sources, each food information source was used in the analysis as a dichotomous variable (used as an information source: yes or no). An 11th category “no information source” was used when respondents indicated using no information sources (No on all information sources), this group was not taken up as an independent variable in the analyses. Finally, to test the internal reliability, the first 100 surveys filled in were categorized by 2 researchers independently. The coding results were similar, with a Cronbach alpha of at least 0.68 and being above 0.89 in 8 of the 11 categories.

**TABLE 2:** Coding scheme to classify food information sources (association between media and diet, Belgium, 2022).

Code	Used when respondents mentioned
Celebrity chefs	Names, books or TV shows from celebrity chefs
Family and acquaintances	Family or acquaintances as their main source of influence
Lifestyle gurus	Names, books, or sites from lifestyle gurus as a main source of influence
Experts	People with a degree in health or nutrition e.g. a dietician or doctor or when respondents mentioned a governmental institution or a non-governmental organization like “stand up against cancer”
Online media	An online source that could not be further specified to a certain celebrity chef, lifestyle guru or expert
Magazines	A specific magazine
Supermarkets	A supermarket leaflet or the supermarket itself
Traditional sources	Traditional cookbooks or organizations like the farmers union
TV channels	A TV channel without further specification to a show
Others	An inspiration source that could not be categorized under one of the previous codes, for example, meal boxes
No influence	Indicated no source of influence

The number of times each food information source was mentioned served as a measure of its popularity. Furthermore, the point-biserial correlation, rank-biserial correlation or the phi score (in case of two dichotomous variables) was measured between each food information source (e.g., obtaining information from a celebrity chef yes or no) and the sample characteristics BMI, age, education, and sex (male/female) was measured to gain further insight into the characteristics of the followers of each food information source [36].

Logistic regression was used to measure the association between food information sources used and having certain dietary restrictions. The variables resulting from the coding analysis of the food information sources, with the exception of “no information source” (see Table 2) served as the independent variables in the model. Dieting, the avoidance of certain food components, fat, sugar, dairy, or carbohydrates were used as dependent variables. Age, sex, BMI, education, food allergies, intolerances, and restrictions regarding animal products (e.g., vegetarian, flexitarian) were taken in as covariates considering their effect on dietary behavior.

Ordinal regression was used to measure the association between the use of different food information sources and dietary intake. All food information sources (see Table 2) served as independent variables. Age, sex, BMI, education, food allergies, intolerances and restrictions regarding animal products (e.g., vegetarian, flexitarian), and dieting were used as covariates. The effect sizes were interpreted using odds ratios (OR). As is the case for logistic regression, the odds ratio in ordinal regression reflects an overall change in odds for a unit increase in the dependent variable after a level change in the independent variable [37]. In case of this study, an OR = 2 should be interpreted as a one unit increase in dietary intake being two times more likely when the respondent does (vs. does not) use the respective food medium. While odds ratios within the different ordinal categories may deviate randomly from the overall odds ratio, the overall estimate is valid [37]. In all tests, variables were considered significant if the *p-value* was below 0.05. All analyses were conducted using SPSS version 25.

## Results

Table 3 shows the popularity of all food information. Celebrity chefs were the most popular, with 37% of respondents using them as their main source of food information. Family and acquaintances (21%) came in second, followed by lifestyle gurus (12%), online sources (7%), experts (6%), magazines (5%), supermarkets (5%), traditional cookbooks and organizations (5%), and TV channels (5%). Furthermore, 21% of the respondents reported they did not use any source of food information. Notably, following lifestyle gurus was correlated with being female (r = 0.16, *p <* 0.001), having a higher education (r = 0.12, *p <* 0.001), and being younger (r = −0.07, *p* = 0.01). Similarly, there was a correlation between following celebrity chefs and being female (r = 0.07, *p* = 0.015). A more extensive correlation matrix is presented in Supplementary Appendix S2.

**TABLE 3:** The relation between popular food media information sources and dietary restrictions (*n* = 1115) (association between media and diet, Belgium, 2022).

	Lifestyle gurus			Celebrity chefs			Experts		
	Odds ratio	95% confidence interval	*p-value*	Odds ratio	95% confidence interval	*p-value*	Odds ratio	95% confidence interval	*p-value*
Follows a diet	1.24	0.61–2.51	0.56	0.65	0.35–1.21	0.17	3.28	1.60–6.75	0.001*
Avoids certain food components	2.90	1.85–4.54	<0.001*	0.81	0.55–1.19	0.28	4.10	2.32–7.26	<0.001*
Avoids carbohydrates	2.55	1.21–5.37	0.013*	0.89	0.43–1.85	0.75	2.22	0.84–5.84	0.11
Avoids sugar	2.53	1.59–4.04	<0.001*	0.97	0.64–1.47	0.88	2.53	1.45–4.41	<0.001*
Avoids fat	2.53	1.48–4.33	<0.001*	0.88	0.55–1.40	0.58	2.23	1.21–4.12	0.011*
Avoids dairy	2.64	1.01–6.90	0.048*	0.71	0.29–1.78	0.47	1.06	0.28–4.05	0.93

*Variable has a significant effect (*p* < 0.05).

Notes: logistic regression was used to examine the association between different sources of food information and dietary restrictions. Age, sex, education, other food information sources, BMI, food allergies, intolerances and restrictions regarding animal products (e.g., vegetarian, flexitarian) were taken in as covariates. A more extensive version of Table 3 can be found in Supplementary Appendix S3.

Table 3 shows the association between using different food information sources and dietary restrictions. In total, 8.1% of the respondents were on a diet, while 36% were avoiding certain food components.

Table 3 shows that respondents who used lifestyle gurus as a main source of food information had the most dietary restrictions. They were more likely to avoid carbohydrates (OR = 2.55, 95% CI = 1.21–5.37), sugar (OR = 2.53, 95% CI = 1.59–4.04, fat (OR = 2.53, 95% CI = 1.48–4.33, and dairy (OR = 2.64, 95% CI = 1.01–5.37) (all *p* < 0.05) (see Table 2). People who use experts as an information source were more likely to follow a diet (OR = 3.28, 95% CI = 1.60–6.75, *p* = 0.001) and to restrict certain food components (OR = 4.10, 95% CI = 1.60–6.75, *p* < 0.001) in general. They also showed an odds ratio of 2.53 (95% CI = 1.45–4.41) and 2.23 (95% CI = 1.21–4.12) to avoid sugar, and avoid fat (all *p* < 0.05). In contrast, the use of supermarkets, online sources, magazines, tv channels, traditional sources, and celebrity chefs was not associated with any dietary restrictions (Supplementary Appendix S3).

Table 4 shows that respondents who turn to lifestyle gurus for information consumed fruits, vegetables, legumes, processed and unprocessed meat replacers, plant-based dairy alternatives, nuts and seeds, light soda, cereal, and fish more frequently (all OR’s ranging from 1.45 to 2.36, all *p’s* < 0.05). Among these vegetables, nuts and seeds and plant based dairy showed an OR higher than 2. They also consumed soda, processed red meat, white bread, and potatoes (all OR’s ranging from 0.61 to 0.72, all *p* < 0.05) less often. Respondents who use celebrity chefs as an information source ate vegetables, soda, white bread, water, pastry, cereal, cheese, milk-based drinks, semi-skimmed and skimmed milk, processed and non-processed red meat, non-processed white meat, and potatoes more often (all OR = 1.29–1.53, all *p’s* < 0.05). Respondents relying on experts for information consumed fruit, processed and unprocessed meat replacers, yoghurt, nuts, and seeds more often (all OR’s ranging from 1.73–2.19, all *p’s* < 0.05), while consuming semi-skimmed and skimmed milk and potatoes less often (all OR’s ranging from 0.59–0.62, all *p’s* < 0.05). The usage of other food information sources relates only to the intake frequency of 3 food groups or fewer.

**TABLE 4:** The relation between popular food media information sources and dietary intake (*n* = 1115) (association between media and diet, Belgium, 2022).

	Lifestyle gurus			Celebrity chefs			Experts		
	Odds ratio	95% confidence interval	*p-value*	Odds ratio	95% confidence interval	*p-value*	Odds ratio	95% confidence interval	*p-value*
Candies	1.07	0.76–1.51	0.69	1.07	0.85–1.35	0.58	0.67	0.44–1.03	0.07
Cereal	1.45	1.03–2.04	0.03*	1.29	1.02–1.64	0.04*	1.25	0.78–2.00	0.36
Cheese	1.13	0.80–1.60	0.48	1.45	1.15–1.84	0.002*	1.52	0.97–2.39	0.09
Crisps	0.87	0.62–1.24	0.44	1.25	0.98–1.59	0.07	0.76	0.48–1.19	0.23
Energy drinks	0.6	0.35–1.01	0.06	1.33	0.96–1.85	0.09	0.67	0.33–1.37	0.27
Fish	1.47	1.05–2.07	0.03*	1.15	0.91–1.46	0.24	1.47	0.96–2.26	0.08
Fries	0.92	0.65–1.32	0.67	1.26	0.98–1.61	0.07	0.94	0.60–1.49	0.8
Fruit	1.89	1.31–2.73	<0.001*	1.03	0.81–1.31	0.83	2.17	1.32–3.58	0.001*
Legumes	1.57	1.11–2.22	0.01*	1.2	0.95–1.51	0.13	1.13	0.74–1.74	0.57
Light soda	1.48	1.06–2.06	0.02*	1.23	0.97–1.56	0.09	1.07	0.70–1.65	0.75
Milk based drinks	0.72	0.47–1.11	0.13	1.36	1.02–1.82	0.03*	1.21	0.72–2.05	0.47
Non proc. red meat	0.83	0.59–1.16	0.27	1.39	1.10–1.75	0.01*	0.98	0.63–1.53	0.93
Non proc. white meat	1.28	0.91–1.80	0.162	1.31	1.03–1.66	0.03*	1.15	0.74–1.79	0.54
Nuts and seeds	2.36	1.69–3.30	<0.001*	1.23	0.97–1.55	0.86	1.83	1.18–2.84	0.007*
Pastry	0.99	0.71–1.40	0.97	1.53	1.21–1.94	<0.001*	0.89	0.57–1.38	0.61
Plant based dairy	2.19	1.54–3.11	<0.001*	1.26	0.97–1.63	0.88	1.31	0.80–2.15	0.28
Potatoes	0.69	0.49–0.97	0.03*	1.37	1.08–1.73	0.005*	0.59	0.38–0.92	0.02*
Proc. meat replacers	1.65	1.02–2.16	0.007*	1.14	0.78–1.37	0.34	1.78	1.05–2.85	0.02*
Proc. red meat	0.65	0.46–0.92	0.02*	1.51	1.19–1.91	0.002*	0.89	0.57–1.37	0.59
Proc. white meat	0.9	0.64–1.28	0.56	0.96	0.76–1.21	0.73	0.99	0.64–1.55	0.98
Skimmed/semi-skimmed milk	1.11	0.80–1.54	0.53	1.39	1.10–1.76	0.01*	0.62	0.39–0.98	0.04*
Soda	0.62	0.44–0.89	0.009*	1.3	1.02–1.64	0.03*	0.75	0.47–1.20	0.23
Unproc. meat replacers	1.48	1.02–2.16	0.04*	1.03	0.78–1.37	0.83	1.73	1.05–2.85	0.03*
Vegetables	2.36	1.54–3.62	<0.001*	1.42	1.09–1.86	0.009*	1.22	0.73–2.05	0.44
Water	1.49	0.85–2.61	0.17	1.51	1.07–2.13	0.02*	1.88	0.89–3.96	0.1
White bread	0.61	0.43–0.86	0.005*	1.44	1.14–1.83	0.003*	0.93	0.60–1.45	0.75
Whole grain bread	1.34	0.93–1.92	0.12	1.03	0.81–1.32	0.8	1.04	0.64–1.68	0.89
Whole milk	0.95	0.65–1.37	0.76	1.11	0.86–1.43	0.43	0.89	0.55–1.45	0.64
Yoghurt	1.4	0.99–1.97	0.05	1.16	0.92–1.46	0.21	1.73	1.10–2.71	0.02*

*Variable has a significant effect (*p* < 0.05).

Note: ordinal regression was used to examine the association between food information sources and dietary intake. Age, sex, education other food information sources, BMI, food allergies, intolerances and restrictions regarding animal products (e.g., vegetarian, flexitarian) were taken in as covariates. A more extensive version of Table 4 can be found in Supplementary Appendix S4.

## Discussion

In light of the wide availability of food information and the increased availability of mass media, a survey among a sample of the Flemish adult population was set up to measure the most popular food information sources and their relations with dietary intake and dietary restrictions.

Most people in this study mentioned celebrity chefs, family or acquaintances, and lifestyle gurus as their most popular source of food information. Following celebrity chefs was correlated with being male. This is in line with Taillie [38], who reported an association between the rise of celebrity chefs and the number of men cooking at home. Being female and having a higher education was associated with obtaining advice from lifestyle gurus. A plausible explanation is a greater concern around healthy eating, fear of gaining weight and a poorer self-body image, which are main motivators for adhering to fad diets [39, 40]. Notably, only 6% of the respondents mentioned experts, primarily doctors and dieticians, as their main source of food information. This further supports the idea of people turning to media for food information, while experts, who have smaller media platforms, are losing their role as gatekeeper [8, 41].

As for dietary restrictions, 8.1% of the respondents were on a diet. Experts were the only food information source associated with dieting. Besides the number of respondents following a specific diet, 36% of the respondents reported avoiding certain food components. As suggested by Byrne et al. [27], having dietary restrictions was associated with using lifestyle gurus as a main source of food information, but also with using experts. Using lifestyle gurus as a main source of information was specifically associated with avoiding carbohydrates, sugar, fat, and dairy, while respondents who used experts as a main source were more likely to avoid sugar and fat. The rather low number of dieters and rather high avoidance of food components among people who turn to lifestyle gurus could be attributed to the tendency of lifestyle gurus to not position their guidelines as a “diet.” As the saying goes, “it is not a diet, it is a lifestyle.” The two most popular Flemish lifestyle gurus, Sandra Bekkari and Pascale Naessens, indeed express their detest for the word diet by publishing books called “ik haat diëten” (I hate dieting) and “nooit meer diëten” (never diet again).

The extent to which the use of specific food sources was associated with the intake frequency of different food groups varies. Lifestyle gurus show the greatest relation, as using them as a main source of food information was associated with the intake frequency of 14 food categories and the highest OR among these categories. The most popular source, celebrity chefs, was associated with 13 food categories. Experts came in third, showing an association with seven different food categories. Other information source categories only showed an association with three or fewer categories.

The high number of associations and higher OR with lifestyle gurus has multiple explanations. A first could be the “guru effect.” According to this theory, people tend to “judge profound what they failed to grasp” [42]. Considering the confusion many people have regarding healthy food, they can perceive rather obscure opinions of lifestyle gurus as a sign of intelligence [41]. In contrast to experts, whose ideas and guidelines change over time because of new findings, gurus are more likely to stick to their own idea and provide certainty [41]. Additionally, Baker and Rojek [26] also noted that the relationship between lifestyle gurus and their audience is an important factor [26]. Lifestyle gurus are relatable, they talk about the same struggles and experiences as normal people but also seem to excel in their roles, making them exemplary. Furthermore, Schouten et al. [28] reported a difference in “identification with” and “credibility” as the reason why the endorsement of influencers (lifestyle gurus) is more influential than that of celebrities. Similarly, Zopiatos and Melanthiou [13] also attributed the influence of celebrity chefs to the observation that consumers relate to them and consider them to be trustworthy.

When looking at the specific food groups lifestyle gurus was not only associated with a more frequent consumption of vegetables, fruit, light soda, cereal, nuts, seeds, processed and unprocessed meat replacers, plant-based dairy alternatives, fish, and legumes, but also with less frequent consumption of soda, potatoes, processed red meat, and white bread. Considering that the general population in Flanders eats too few legumes, fruit, vegetables, nuts, seeds, and whole grains, while consuming too much meat, it implicates lifestyle gurus may promote more healthy dietary choices [43, 44]. Similarly, using experts as an information source was associated with more frequent consumption of fruit, processed and unprocessed meat-replacers, yoghurt, nuts, and seeds as well as the less frequent consumption of potatoes, as well as semi-skimmed and skimmed milk. While dairy is still included in Belgian dietary guidelines, some studies suggest that replacing dairy with nuts and other plant based sources would have a beneficial effect on cardiovascular and all-cause mortality [45, 46]. Similarly, as the Flemish population consumes too much meat, meat replacers have been suggested as a healthy and more environmentally friendly alternative [47]. However, processed meat substitutes often score very high on sodium and sugar rendering also these products less healthy [48, 49]. It is also notable that potatoes were eaten less often by both those who report following experts and those following lifestyle gurus. According to the Belgian nutritional guidelines, potatoes can be part of a healthy diet. However, other sources (e.g., the nutritional guidelines from Harvard) suggest potatoes possess too few vitamins and a too-high glycemic index [50].

Respondents who turned to chefs as information sources ate vegetables, soda, water, white bread, pastry, cereal, cheese, milk based drinks, semi-skimmed and skimmed milk, processed and non-processed red meat, non-processed white meat, and potatoes more often. The OR were however, smaller compared to the OR of lifestyle gurus and experts. The frequent use of potatoes in the traditional Belgian kitchen could explain the high potato consumption in these groups. Additionally, high pastry consumption could reflect the increasing popularity of baking shows like “Bake Off.” Increases in the consumption frequency of vegetables, white bread, meat, and cheeses were likely a result of their frequent use in the recipes of chefs. As using celebrity chefs as a food information source had a positive association with the intake frequency of food groups like water and vegetables which are consumed too infrequently, but also with white bread, pastry, processed red meat which are consumed too frequently, it is not possible to conclude whether it associates with a more or less balanced dietary intake [34].

Considering the criticism from the scientific community on diets endorsed by lifestyle gurus, it is rather surprising that the results show a positive relation between following lifestyle gurus and dietary components that are consumed insufficiently among the general population. However, it is notable that most popular lifestyle gurus referenced in this questionnaire strive toward consuming less processed foods, which is indeed beneficial considering the association processed food consumption has with obesity, overall cancer, all-cause mortality, cardiovascular diseases and depression [51, 52]. The restriction against carbohydrates, fat, and dairy is more controversial, as dietary restrictions or “clean eating” is often associated with eating disorders [53, 54]. Similarly the focus on restricting macronutrients has shown only limited effect on weight control, while putting people at risk for nutritional deficiencies, alterations in the gut microbiota and metabolic side-effects [54–57]. With food media becoming increasingly popular, this study underlines the need for more research on the influence food media has on diet.

### Limitations and Implications for Future Research

This study has several limitations. First, although this study had a rather large sample, the study is not entirely representative of the Flemish population: The sample contained relatively more obese but fewer overweight people and more older and lower educated respondents compared to the general Flemish population. Furthermore, the sample characteristics of the respondents who cook daily may differ significantly from the national average. A second limitation is the cross-sectional design. This makes it only possible to show associations, as it is not, for example, possible to know whether following lifestyle gurus leads to dietary restrictions or the other way around. A third limitation is that the SFFQ has its limitations, as it relies on self-reporting and as it measures consumption frequency but not serving size, it could cause an overestimation of dietary intake [33, 58]. Its focus on individual food groups also makes it harder to make statements regarding total diet quality.

For future research, it would be useful to conduct more experimental studies to gain insight in a possible causal relation between food media and dietary intake. Furthermore, it would be interesting to look whether food media icons, like celebrity chefs and lifestyle gurus can be used in the promotion of healthier lifestyles.

### Conclusion

The findings underline the role of media as gatekeeper of the population’s diets. Respondents perceive celebrity chefs, family and acquaintances, and lifestyle gurus as their main source of food information, ahead of experts and governments. Using these Food media sources was associated with dietary intake of specific food groups. That is, following lifestyle gurus had the strongest associations, and obtaining information from them associated with a more plant-based diet and more dietary restrictions. Relying on celebrity chefs for food information positively correlated with dietary intake of food groups included in their recipes. This study therefore underlines the relation between media and dietary behavior and the need for evidence-based food information to improve the diet quality of the general population.

## References

[1] Haque M. Fresh Food Is in Struggle with Processed: A Global Consternation. Adv Hum Biol (2021) 11(2):200. 10.4103/aihb.aihb_25_21

[2] Popkin BM Adair LS Ng SW. Global Nutrition Transition and the Pandemic of Obesity in Developing Countries. Nutr Rev (2012) 70(1):3–21. 10.1111/j.1753-4887.2011.00456.x 22221213 PMC3257829

[3] WHO. Obesity and Overweight. [Internet] (2021). Available at: https://www.who.int/news-room/fact-sheets/detail/obesity-and-overweight (Accessed January 20, 2020).

[4] Eurostat. Over Half of Adults in the EU Are Overweight. [Internet] (2021). Available at: https://ec.europa.eu/eurostat/web/products-eurostat-news/-/ddn-20210721-2 (Accessed February 8, 2022).

[5] Christ A Lauterbach M Latz E. Western Diet and the Immune System: An Inflammatory Connection. Immunity (2019) 51(5):794–811. 10.1016/j.immuni.2019.09.020 31747581

[6] Yusefzadeh H Rahimi B Rashidi ALI. Economic Burden of Obesity: A Systematic Review. Soc Health Behav (2019) 2:7–12. 10.4103/SHB.SHB_37_18

[7] OECD. The Heavy Burden of Obesity: The Economics of Prevention. [Internet]. Paris: Organisation for Economic Co-operation and Development (2019). Available at: https://www.oecd-ilibrary.org/social-issues-migration-health/the-heavy-burden-of-obesity_67450d67-en (Accessed November 4, 2021).

[8] Boylan S Louie JCY Gill TP. Consumer Response to Healthy Eating, Physical Activity and Weight-Related Recommendations: a Systematic Review. Obes Rev (2012) 13(7):606–17. 10.1111/j.1467-789x.2012.00989.x 22404752

[9] Korthals M. Ethics of Dietary Guidelines: Nutrients, Processes and Meals. J Agric Environ Ethics (2017) 30(3):413–21. 10.1007/s10806-017-9674-7

[10] Kosonen H Rimpelä A Rauma AL Väisänen P Pere L Virtanen SM Consumption of Special Diet Among Finnish Adolescents in 1979-2001: Repeated National Cross-Sectional Surveys. Soz.-Präventivmed. (2005) 50(3):142–50. 10.1007/s00038-005-3167-6 16010813

[11] Pollard CM Pulker CE Meng X Kerr DA Scott JA. Who Uses the Internet as a Source of Nutrition and Dietary Information? An Australian Population Perspective. J Med Internet Res (2015) 17(8):e209. 10.2196/jmir.4548 26310192 PMC4642382

[12] De Backer CJS Hudders L. Look Who's Cooking. Investigating the Relationship between Watching Educational and Edutainment TV Cooking Shows, Eating Habits and Everyday Cooking Practices Among Men and Women in Belgium. Appetite. (2016) 96:494–501. 10.1016/j.appet.2015.10.016 26485291

[13] Zopiatis A Melanthiou Y. The Celebrity Chef Phenomenon: a (Reflective) Commentary. Int. J. Contemp. Hosp. Manag. (2019) 31(2):538–56. 10.1108/ijchm-12-2017-0822

[14] Howard S Adams J White M. Nutritional Content of Supermarket Ready Meals and Recipes by Television Chefs in the United Kingdom: Cross Sectional Study. BMJ (2012) 345:e7607. 10.1136/bmj.e7607 23247976 PMC3524368

[15] Jones M Freeth EC Hennessy-Priest K Costa RJS. A Systematic Cross-Sectional Analysis of British Based Celebrity Chefs’ Recipes: Is There Cause for Public Health Concern? Food Public Health Maart (2013) 3(2):100–10. 10.5923/fph.20130302.04

[16] Trattner C Elsweiler D Howard S. Estimating the Healthiness of Internet Recipes: A Cross-Sectional Study. Front Public Health (2017) 5:16. 10.3389/fpubh.2017.00016 28243587 PMC5304340

[17] Bonaccio M Di Castelnuovo A Costanzo S De Lucia F Olivieri M Donati MB Mass media Information and Adherence to Mediterranean Diet: Results from the Moli-Sani Study. Int J Public Health (2012) 57(3):589–97. 10.1007/s00038-011-0327-8 22187041

[18] Lane SR Fisher SM. The Influence of Celebrity Chefs on a Student Population. Br Food J (2015) 117(2):614–28. 10.1108/bfj-09-2013-0253

[19] Pope L Latimer L Wansink B. Viewers vs. Doers. The Relationship between Watching Food Television and BMI. Appetite. (2015) 90:131–5. 10.1016/j.appet.2015.02.035 25747286

[20] Clifford D Anderson J Auld G Champ J. Good Grubbin': Impact of a TV Cooking Show for College Students Living off Campus. J Nutr Edu Behav (2009) 41(3):194–200. 10.1016/j.jneb.2008.01.006 19411053

[21] Villani AM Egan T Keogh JB Clifton PM. Attitudes and Beliefs of Australian Adults on Reality Television Cooking Programmes and Celebrity Chefs. Is There Cause for Concern? Descriptive Analysis Presented from a Consumer Survey. Appetite. (2015) 91:7–12. 10.1016/j.appet.2015.03.021 25819603

[22] Clarke TB Murphy J Adler J. Celebrity Chef Adoption and Implementation of Social Media, Particularly Pinterest: A Diffusion of Innovations Approach. Int J Hosp Manag (2016) 57:84–92.

[23] Elias MJ. Food on the page: Cookbooks and American Culture. Philadelphia: University of Pennsylvania Press (2017).

[24] Baker SA Rojek C. Lifestyle Gurus: Constructing Authority and Influence Online. John Wiley & Sons (2020):13

[25] Stanton R. Diet Gurus Ignore the Weight of Evidence in Guidelines. Australas Sci (2018) 39(4):44.

[26] Baker SA Rojek C. The Belle Gibson Scandal: The Rise of Lifestyle Gurus as Micro-celebrities in Low-Trust Societies. J Sociol (2019) 10:1440783319846188. 10.1177/1440783319846188

[27] Byrne E Kearney J MacEvilly C. The Role of Influencer Marketing and Social Influencers in Public Health. Proc Nutr Soc (2017) 76(OCE3):103. 10.1017/s0029665117001768

[28] Schouten AP Janssen L Verspaget M. Celebrity vs. Influencer Endorsements in Advertising: the Role of Identification, Credibility, and Product-Endorser Fit. Int J Advert (2019) 39(2):1–24. 10.1080/02650487.2019.1634898

[29] Dickinson K Watson M Prichard I. Are Clean Eating Blogs a Source of Healthy Recipes? A Comparative Study of the Nutrient Composition of Foods with and without Clean Eating Claims. Nutrients. (2018) 10(10):1440. 10.3390/nu10101440 PMC6213725 30301131

[30] Schneider EP McGovern EE Lynch CL Brown LS. Do Food Blogs Serve as a Source of Nutritionally Balanced Recipes? An Analysis of 6 Popular Food Blogs. J Nutr Edu Behav (2013) 45(6):696–700. 10.1016/j.jneb.2013.07.002 24206585

[31] Drieskens S Gieskens L Charafeddine R Demares S Braekman E Nguyen D Gezondheidsenquête 2018. [Internet]. Sciensano (2018). p. 52. Available at: https://his.wiv-isp.be/nl/Gedeelde%20%20documenten/summ_LS_NL_2018.pdf (Accessed January 25, 2021).

[32] Statistiek Vlaanderen. Bevolking Naar Onderwijsniveau (Scholingsgraad). [Internet] (2019). Available at: /nl/bevolking-naar-onderwijsniveau-scholingsgraad (Accessed January 25, 2021).

[33] Vereecken CA Maes L. A Belgian Study on the Reliability and Relative Validity of the Health Behaviour in School-Aged Children Food-Frequency Questionnaire. Public Health Nutr (2003) 6(6):581–8. 10.1079/phn2003466 14690039

[34] Gezond leven. Nieuwe Adviezen Van Hoge Gezondheidsraad Bevestigen Voedingsdriehoek. [Internet]. Vlaams Instituut Gezond Leven (2019). Available at: https://www.gezondleven.be/nieuws/nieuwe-adviezen-van-hoge-gezondheidsraad-bevestigen-voedingsdriehoek (Accessed January 21, 2020).

[35] Jáuregui-Lobera I. Fad Diets, Miracle Diets, Diet Cult. but No Results. JONNPR (2017) 2(3):90–3.

[36] Brown JD. Point-biserial Correlation Coefficients. Statistics (2001) 5(3):12–6.

[37] Senn S Julious S. Measurement in Clinical Trials: a Neglected Issue for Statisticians? Statist Med (2009) 28(26):3189–209. 10.1002/sim.3603 19455540

[38] Taillie LS. Who's Cooking? Trends in US home Food Preparation by Gender, Education, and Race/ethnicity from 2003 to 2016. Nutr J (2018) 17:41. 10.1186/s12937-018-0347-9 29609591 PMC5881182

[39] Banjari I Kenjerić D Mandić ML. Is Fad Diet a Quick Fix? An Observational Study in a Croatian Student Group. Period Biol (2011) 113(3):377–81. 10.1002/sim.3603

[40] Slof-Op 't Landt MCT van Furth EF van Beijsterveldt CEM Bartels M Willemsen G de Geus EJ Prevalence of Dieting and Fear of Weight Gain across Ages: A Community Sample from Adolescents to the Elderly. Int J Public Health (2017) 62(8):911–9. 10.1007/s00038-017-0948-7 28220234

[41] Garza C Stover PJ Ohlhorst SD Field MS Steinbrook R Rowe S Best Practices in Nutrition Science to Earn and Keep the Public's Trust. Am J Clin Nutr (2019) 109(1):225–43. 10.1093/ajcn/nqy337 30657846 PMC6900562

[42] Sperber D. The Guru Effect. Rev.Phil.Psych. (2010) 1(4):583–92. 10.1007/s13164-010-0025-0

[43] Drieskens S. Voedingsgewoonten (Gezondheidsenquête 2018). [Internet]. Sciensano (2018). p. 144. Available at: https://his.wiv-isp.be/nl/Gedeelde%20%20documenten/summ_LS_NL_2018.pdf (Accessed February 3, 2021).

[44] Gezond leven. Wat eet de gemiddelde Vlaming? [Internet] (2020). Available at: https://www.gezondleven.be/themas/voeding/cijfers/wat-eet-de-gemiddelde-vlaming (Accessed February 3, 2021).

[45] Willett W Rockström J Loken B Springmann M Lang T Vermeulen S Food in the Anthropocene: the EAT-Lancet Commission on Healthy Diets from Sustainable Food Systems. Lancet (2019) 393(10170):447–92. 10.1016/s0140-6736(18)31788-4 30660336

[46] Ding M Li J Qi L Ellervik C Zhang X Manson JE Associations of Dairy Intake with Risk of Mortality in Women and Men: Three Prospective Cohort Studies. BMJ (2019) 367:l6204. 10.1136/bmj.l6204 31776125 PMC6880246

[47] Mertens E Biesbroek S Dofková M Mistura L D’Addezio L Turrini A Potential Impact of Meat Replacers on Nutrient Quality and Greenhouse Gas Emissions of Diets in Four European Countries. Sustainability (2020) 12(17):6838. 10.3390/su12176838

[48] Farsi DN Uthumange D Munoz Munoz J Commane DM. The Nutritional Impact of Replacing Dietary Meat with Meat Alternatives in the UK: A Modelling Analysis Using Nationally Representative Data. Br J Nutr (2021) 1–11. 10.1017/s0007114521002750 PMC9201833 34284832

[49] Curtain F Grafenauer S. Plant-based Meat Substitutes in the Flexitarian Age: An Audit of Products on Supermarket Shelves. Nutrients (2019) 11(11):2603. 10.3390/nu11112603 PMC6893642 31671655

[50] Harvard T. H. Chan. The Nutrition Source. Healthy Eating Plate. [Internet] (2011). Available at: https://www.hsph.harvard.edu/nutritionsource/healthy-eating-plate/ (Accessed January 21, 2020).

[51] Chen X Zhang Z Yang H Qiu P Wang H Wang F Consumption of Ultra-processed Foods and Health Outcomes: a Systematic Review of Epidemiological Studies. Nutr J (2020) 19(1):86. 10.1186/s12937-020-00604-1 32819372 PMC7441617

[52] Pagliai G Dinu M Madarena MP Bonaccio M Iacoviello L Sofi F. Consumption of Ultra-processed Foods and Health Status: a Systematic Review and Meta-Analysis. Br J Nutr (2021) 125(3):308–18. 10.1017/s0007114520002688 32792031 PMC7844609

[53] Heaney RP. Dairy Intake, Dietary Adequacy, and Lactose Intolerance. Adv Nutr (2013) 4(2):151–6. 10.3945/an.112.003368 23493531 PMC3649095

[54] Ambwani S Shippe M Gao Z Austin SB. Is #cleaneating a Healthy or Harmful Dietary Strategy? Perceptions of Clean Eating and Associations with Disordered Eating Among Young Adults. J Eat Disord (2019) 7(1):17. 10.1186/s40337-019-0246-2 31171970 PMC6545628

[55] Freedhoff Y Hall KD. Weight Loss Diet Studies: We Need Help Not Hype. Lancet. (2016) 388(10047):849–51. 10.1016/s0140-6736(16)31338-1 27597452

[56] Cordain L. Nutritional Deficiencies of Ketogenic Diets. Fort Collins, CO: Colorado State University. (2018).

[57] Seganfredo FB Blume CA Moehlecke M Giongo A Casagrande DS Spolidoro JVN Weight-loss Interventions and Gut Microbiota Changes in Overweight and Obese Patients: a Systematic Review. Obes Rev (2017) 18(8):832–51. 10.1111/obr.12541 28524627

[58] Vereecken CA Rossi S Giacchi MV Maes L. Comparison of a Short Food-Frequency Questionnaire and Derived Indices with a Seven-Day Diet Record in Belgian and Italian Children. Int J Public Health (2008) 53(6):297–305. 10.1007/s00038-008-7101-6 19112592

