# Supplementary material for: Food Media and Dietary Behavior in a Belgian Adult Sample: How Obtaining Information From Food Media Sources Associates With Dietary Behavior
Source: Int J Public Health. 2022 May 23;67:1604627. doi: 10.3389/ijph.2022.1604627 (PMC9169530; doi:10.3389/ijph.2022.1604627)
Supplement: Supplementary file 1 [file DataSheet1.docx]

Appendix 1: additional items added to the Short Food Frequency Questionnaire [association between media and diet, Belgium, 2022]

|  | never | less than once a week/seldom | once a week | 2-4 days a week | 5-6 days a week | every day | more than once a day |
| --- | --- | --- | --- | --- | --- | --- | --- |
| charcuterie |  |  |  |  |  |  |  |
| legumes, (e.g. chickpeas, beans, lentils, fresh or tinned) |  |  |  |  |  |  |  |
| nuts and seeds (unsalted, without sweet or salt coating) |  |  |  |  |  |  |  |
| plant-based dairy alternatives (e.g. soymilk, oat drinks, soya yogurt) |  |  |  |  |  |  |  |
| potatoes |  |  |  |  |  |  |  |
| processed meat replaces (e.g. vegetarian burgers, Quorn, pre-made vegetarian spreads) |  |  |  |  |  |  |  |
| processed red meat (e.g. sausages, bacon, burgers) |  |  |  |  |  |  |  |
| processed white meat (e.g. Chicken nuggets, chicken salami) |  |  |  |  |  |  |  |
| unprocessed meat replaces (e.g. tempeh, seitan, tofu) |  |  |  |  |  |  |  |
| unprocessed red meat (e.g. fresh pork, fresh lamb) |  |  |  |  |  |  |  |
| unprocessed white meat (e.g. chicken breast, poultry) |  |  |  |  |  |  |  |

Appendix 2: Correlation matrix between food information sources and sample characteristics [association between media and diet, Belgium, 2022]

|  | lifestyle gurus | celebrity chef | family and acquiantances | experts | online media | supermarket | magazine | TV channels | other food information sources | traditional sources |
| --- | --- | --- | --- | --- | --- | --- | --- | --- | --- | --- |
| gender^a^ (0 =male, 1=female) | 0.16^*^ | -0.07^*^ | -0.02 | -0.01 | 0.08^*^ | -0.01 | 0.13^**^ | -0.07^*^ | -0.04 | 0.03 |
| age^b^ | -0.07^*^ | 0.00 | -0.03 | 0.05 | -0.14^*^ | -0.03 | 0.00 | -0.09^*^ | -0.07* | 0.12^*^ |
| Education^c^ | 0.12^*^ | 0.06 | 0.03 | 0.03 | 0.01 | 0.04 | 0.07^*^ | 0.02 | 0.03 | 0.01 |
| BMI^b^ | -0.02 | 0.06 | -0.01 | 0.04 | -0.06 | -0.01 | 0.02 | 0.01 | 0.02 | 0.06* |

* Variable shows a significant correlation (*p*<0.05)
^a^The phi score was measured considering 2 dichotomous variables
^b^The point-biserial correlation was measured considering 1 dichotomous and 1 ratio or interval score
*^c^* The rank-biserial correlation was measured considering 1 dichotomous and 1 ordinal score

Appendix 3a: the relation between food information sources and dietary restrictions (n=1115) [association between media and diet, Belgium, 2022]

|  | follows a diet | | | avoids certain food components | | | avoids carbohydrates | | |
| --- | --- | --- | --- | --- | --- | --- | --- | --- | --- |
|  | OR | 95% C.I. | *p-value* | OR | 95% C.I. | *p-value* | OR | 95% C.I. | *p-value* |
| celebrity chef | 0.65 | 0.35-1.21 | 0.17 | 0.81 | 0.55-1.19 | 0.28 | 0.89 | 0.43-1.85 | 0.75 |
| experts | 3.28 | 1.60-6.75 | 0.001* | 4.1 | 2.32-7.26 | < 0.001* | 2.22 | 0.84-5.84 | 0.11 |
| family and acquaintances | 0.67 | 0.33-1.36 | 0.27 | 0.86 | 0.56-1.32 | 0.49 | 0.29 | 0.09-0.91 | 0.03* |
| lifestyle guru | 1.24 | 0.61-2.51 | 0.56 | 2.9 | 1.85-4.54 | < 0.001* | 2.55 | 1.21-5.37 | 0.013* |
| magazines | 0.44 | 0.14-1.35 | 0.15 | 1.15 | 0.62-2.13 | 0.67 | 0.63 | 0.20-2.00 | 0.43 |
| online media | 1.63 | 0.71-3.74 | 0.25 | 1.62 | 0.93-2.83 | 0.09 | 1.79 | 0.67-4.78 | 0.24 |
| supermarket | 1.32 | 0.47-3.70 | 0.6 | 1.04 | 0.53-2.01 | 0.92 | 1.86 | 0.60-5.72 | 0.28 |
| traditional sources | 0.73 | 0.24-2.28 | 0.59 | 1.5 | 0.80-2.81 | 0.2 | 0.54 | 0.12-2.48 | 0.43 |
| TV channel | 1.86 | 0.70-4.95 | 0.21 | 1.12 | 0.58-2.20 | 0.73 | 2.15 | 0.67-6.90 | 0.2 |
| Other sources | 0.37 | 0.04-3.14 | 0.36 | 0.97 | 0.36-2.58 | 0.95 | 0.91 | 0.10-8.13 | 0.93 |

*Variable has a significant effect (*p*<0.05)
Notes: logistic regression was used to examine the association between different sources of food information and dietary restrictions. Age, sex, education, other food information sources, BMI, food allergies, intolerances and restrictions regarding animal products (e.g. vegetarian, flexitarian) were taken in as covariates.

Appendix 3b: the relation between food information sources and dietary restrictions (n=1115) [association between media and diet, Belgium, 2022]

|  | avoids sugar | | | avoids fat | | | avoids dairy | | |
| --- | --- | --- | --- | --- | --- | --- | --- | --- | --- |
|  | OR | 95% C.I | *p-value* | OR | 95% C.I. | *p-value* | OR | 95% C.I. | *p-value* |
| celebrity chef | 0.97 | 0.64-1.47 | 0.88 | 0.88 | 0.55-1.40 | 0.58 | 0.71 | 0.29-1.78 | 0.47 |
| experts | 2.53 | 1.45-4.41 | <0.001* | 2.23 | 1.21-4.12 | 0.011* | 1.06 | 0.28-4.05 | 0.93 |
| family and acquaintances | 0.75 | 0.46-1.20 | 0.23 | 0.99 | 0.58-1.68 | 0.97 | 0.57 | 0.18-1.83 | 0.35 |
| lifestyle guru | 2.53 | 1.59-4.04 | <0.001* | 2.53 | 1.48-4.33 | <0.001* | 2.64 | 1.01-6.90 | 0.048* |
| magazines | 1.01 | 0.52-1.97 | 0.98 | 0.7 | 0.32-1.55 | 0.38 | 1.42 | 0.36-5.53 | 0.61 |
| online media | 1.51 | 0.82-2.79 | 0.19 | 2.21 | 1.11-4.37 | 0.02* | 2.11 | 0.65-6.88 | 0.21 |
| supermarket | 0.73 | 0.33-1.59 | 0.42 | 1.1 | 0.48-2.55 | 0.82 | 1.79 | 0.48-6.72 | 0.39 |
| traditional sources | 0.93 | 0.46-1.86 | 0.83 | 2.28 | 1.16-4.49 | 0.02* | 1.07 | 0.21-5.38 | 0.93 |
| TV channel | 1.28 | 0.63-2.61 | 0.5 | 1.61 | 0.72-3.61 | 0.25 | 1.33 | 0.28-6.28 | 0.72 |
| Other sources | 1.07 | 0.36-3.19 | 0.91 | / | / | / | 3.55 | 0.59-21.46 | 0.17 |

*Variable has a significant effect (*p*<0.05)
/ nobody avoided this component, therefore no statistical test was possibleNotes: logistic regression was used to examine the association between different sources of food information and dietary restrictions. Age, sex, education, other food information sources, BMI, food allergies, intolerances and restrictions regarding animal products (e.g. vegetarian, flexitarian) were taken in as covariates.

Appendix 4a: The relation between food media information sources and dietary intake (n=1115) [association between media and diet, Belgium, 2022]

|  | familiekennis | | | online | | | supermarkt | | |
| --- | --- | --- | --- | --- | --- | --- | --- | --- | --- |
|  | OR | 95% C.I. | *p-value* | OR | 95% C.I. | *p-value* | OR | 95% C.I. | *p-value* |
| candies | 1.20 | 0.91-1.58 | 0.20 | 0.89 | 0.58-1.36 | 0.59 | 1.93 | 1.16-3.23 | 0.01 |
| cereal | 0.86 | 0.65-1.15 | 0.31 | 1.48 | 0.96-2.29 | 0.08 | 1.37 | 0.82-2.27 | 0.23 |
| cheese | 1.43 | 1.08-1.89 | 0.01 | 1.06 | 0.69-1.62 | 0.80 | 1.66 | 1.02-2.69 | 0.04 |
| crisps | 1.11 | 0.84-1.48 | 0.47 | 1.15 | 0.75-1.77 | 0.53 | 1.63 | 0.99-2.7 | 0.06 |
| energy drinks | 0.93 | 0.63-1.37 | 0.70 | 0.78 | 0.44-1.38 | 0.39 | 0.90 | 0.45-1.8 | 0.76 |
| fish | 0.90 | 0.68-1.19 | 0.45 | 1.30 | 0.83-2.02 | 0.25 | 1.01 | 0.61-1.67 | 0.98 |
| fries | 1.11 | 0.83-1.48 | 0.50 | 1.16 | 0.74-1.83 | 0.53 | 1.11 | 0.64-1.93 | 0.70 |
| fruit | 0.83 | 0.63-1.11 | 0.21 | 1.20 | 0.76-1.89 | 0.44 | 1.03 | 0.62-1.71 | 0.91 |
| legumes | 0.75 | 0.57-0.98 | 0.04 | 1.21 | 0.79-1.84 | 0.38 | 0.83 | 0.52-1.34 | 0.45 |
| light soda | 1.03 | 0.77-1.38 | 0.82 | 0.76 | 0.48-1.2 | 0.24 | 1.03 | 0.61-1.72 | 0.93 |
| milk based drinks | 0.82 | 0.58-1.16 | 0.27 | 0.62 | 0.36-1.09 | 0.10 | 1.12 | 0.61-2.06 | 0.71 |
| non proc. red meat | 0.90 | 0.69-1.19 | 0.47 | 0.79 | 0.51-1.21 | 0.28 | 1.22 | 0.73-2.04 | 0.45 |
| non proc. white meat | 1.12 | 0.85-1.48 | 0.41 | 1.23 | 0.80-1.89 | 0.36 | 1.22 | 0.73-2.04 | 0.44 |
| nuts and seeds | 0.82 | 0.62-1.09 | 0.17 | 1.19 | 0.77-1.83 | 0.44 | 1.41 | 0.87-2.26 | 0.16 |
| pastry | 1.25 | 0.95-1.64 | 0.12 | 1.31 | 0.85-2.01 | 0.23 | 2.49 | 1.51-4.09 | 0.00 |
| plant based dairy | 0.87 | 0.63-1.19 | 0.38 | 1.31 | 0.82-2.09 | 0.26 | 1.32 | 0.78-2.22 | 0.30 |
| potatoes | 1.12 | 0.85-1.48 | 0.42 | 0.96 | 0.63-1.46 | 0.84 | 1.17 | 0.70-1.94 | 0.55 |
| proc. meat replacers | 0.82 | 0.58-1.14 | 0.24 | 1.03 | 0.63-1.68 | 0.90 | 1.66 | 0.98-2.79 | 0.06 |
| proc. red meat | 1.15 | 0.87-1.51 | 0.33 | 0.74 | 0.48-1.15 | 0.18 | 1.41 | 0.84-2.38 | 0.19 |
| proc. white meat | 0.93 | 0.71-1.22 | 0.61 | 0.88 | 0.57-1.35 | 0.55 | 0.96 | 0.59-1.56 | 0.86 |
| skimmed/semi-skimmed milk | 1.19 | 0.90-1.57 | 0.22 | 1.71 | 1.11-2.62 | 0.02 | 1.47 | 0.90-2.38 | 0.12 |
| soda | 1.12 | 0.85-1.48 | 0.43 | 0.56 | 0.36-0.88 | 0.01 | 0.79 | 0.48-1.32 | 0.37 |
| unproc. meat replacers | 0.76 | 0.54-1.06 | 0.11 | 1.04 | 0.64-1.7 | 0.87 | 1.57 | 0.93-2.66 | 0.09 |
| vegetables | 0.93 | 0.69-1.26 | 0.65 | 1.36 | 0.84-2.18 | 0.21 | 1.62 | 0.91-2.89 | 0.10 |
| water | 0.95 | 0.64-1.38 | 0.77 | 1.18 | 0.63-2.21 | 0.61 | 1.03 | 0.53-2.01 | 0.94 |
| white bread | 1.21 | 0.92-1.59 | 0.18 | 0.85 | 0.54-1.32 | 0.47 | 0.77 | 0.47-1.28 | 0.32 |
| Whole grain bread | 1.26 | 0.94-1.69 | 0.12 | 0.75 | 0.48-1.15 | 0.19 | 1.44 | 0.82-2.53 | 0.20 |
| whole milk | 1.00 | 0.74-1.35 | 0.98 | 0.68 | 0.42-1.13 | 0.14 | 0.69 | 0.39-1.21 | 0.20 |
| yoghurt | 0.97 | 0.74-1.27 | 0.81 | 0.95 | 0.63-1.43 | 0.80 | 1.03 | 0.63-1.67 | 0.92 |

*Variable has a significant effect (*p*<0.05)
Note: ordinal regression was used to examine the association between food information sources and dietary intake. Age, sex, education other food information sources, BMI, food allergies, intolerances and restrictions regarding animal products (e.g. vegetarian, flexitarian) were taken in as covariates

Appendix 4b: The relation between food media information sources and dietary intake (n=1115) [association between media and diet, Belgium, 2022]

|  | magazine | | | other tv | | | Other food information channels | | |
| --- | --- | --- | --- | --- | --- | --- | --- | --- | --- |
|  | OR | 95% C.I. | *p-value* | OR | 95% C.I. | *p-value* | OR | 95% C.I. | *p-value* |
| candies | 0.96 | 0.60-1.54 | 0.86 | 1.46 | 0.89-2.41 | 0.14 | 0.96 | 0.50-1.86 | 0.91 |
| cereal | 1.08 | 0.83-4.98 | 0.77 | 1.53 | 0.91-2.56 | 0.11 | 2.07 | 1.00-4.31 | 0.05 |
| cheese | 1.28 | 0.77-2.12 | 0.34 | 0.87 | 0.51-1.5 | 0.62 | 1.62 | 0.79-3.35 | 0.19 |
| crisps | 1.72 | 1.05-2.83 | 0.03 | 0.98 | 0.58-1.67 | 0.95 | 1.31 | 0.67-2.61 | 0.43 |
| energy drinks | 0.56 | 0.22-1.43 | 0.22 | 0.68 | 0.32-1.42 | 0.30 | 2.02 | 0.86-4.74 | 0.11 |
| fish | 0.98 | 0.59-1.62 | 0.94 | 1.11 | 0.65-1.9 | 0.70 | 1.20 | 0.57-2.52 | 0.63 |
| fries | 1.16 | 0.69-1.93 | 0.58 | 0.68 | 0.39-1.17 | 0.16 | 0.93 | 0.44-1.95 | 0.85 |
| fruit | 1.00 | 0.59-1.7 | 1.00 | 1.08 | 0.62-1.87 | 0.79 | 0.89 | 0.43-1.84 | 0.75 |
| legumes | 1.00 | 0.61-1.63 | 0.99 | 1.04 | 0.62-1.74 | 0.88 | 1.64 | 0.83-3.24 | 0.16 |
| light soda | 0.95 | 0.57-1.6 | 0.85 | 1.16 | 0.69-1.95 | 0.59 | 0.84 | 0.39-1.79 | 0.65 |
| milk based drinks | 1.73 | 0.96-3.09 | 0.07 | 0.64 | 0.34-1.21 | 0.17 | 1.49 | 0.66-3.39 | 0.34 |
| non proc. red meat | 1.52 | 0.93-2.51 | 0.10 | 0.87 | 0.51-1.46 | 0.59 | 2.40 | 1.14-5.06 | 0.02 |
| non proc. white meat | 1.17 | 0.7-1.94 | 0.55 | 1.13 | 0.66-1.93 | 0.65 | 2.28 | 1.08-4.83 | 0.03* |
| nuts and seeds | 1.13 | 0.70-1.81 | 0.63 | 1.40 | 0.85-2.31 | 0.18 | 2.07 | 1.01-4.25 | 0.05 |
| pastry | 0.96 | 0.59-1.56 | 0.87 | 1.62 | 0.97-2.72 | 0.07 | 1.23 | 0.62-2.46 | 0.55 |
| plant based dairy | 0.88 | 0.50-1.55 | 0.66 | 1.24 | 0.7-2.19 | 0.47 | 1.72 | 0.79-3.73 | 0.17 |
| potatoes | 0.92 | 0.56-1.51 | 0.74 | 0.81 | 0.49-1.33 | 0.40 | 0.94 | 0.47-1.9 | 0.87 |
| proc. meat replacers | 1.55 | 0.91-2.65 | 0.11 | 0.83 | 0.45-1.53 | 0.55 | 1.91 | 0.86-4.23 | 0.11 |
| proc. red meat | 1.56 | 0.92-2.64 | 0.10 | 0.76 | 0.45-1.3 | 0.32 | 2.18 | 1.00-4.77 | 0.05 |
| proc. white meat | 1.38 | 0.84-2.26 | 0.21 | 1.25 | 0.75-2.07 | 0.39 | 1.50 | 0.75-3.02 | 0.26 |
| skimmed/semi-skimmed milk | 1.58 | 0.97-2.56 | 0.06 | 0.63 | 0.39-1.04 | 0.07 | 1.26 | 0.64-2.48 | 0.51 |
| soda | 1.30 | 0.79-2.16 | 0.31 | 0.95 | 0.55-1.61 | 0.84 | 1.35 | 0.68-2.68 | 0.40 |
| unproc. meat replacers | 1.17 | 0.66-2.05 | 0.60 | 1.24 | 0.69-2.21 | 0.48 | 1.78 | 0.80-3.96 | 0.16 |
| vegetables | 1.81 | 0.96-3.4 | 0.07 | 0.83 | 0.47-1.48 | 0.53 | 1.48 | 0.69-3.18 | 0.32 |
| water | 1.39 | 0.57-3.43 | 0.47 | 2.31 | 0.87-6.12 | 0.09 | 1.25 | 0.51-3.07 | 0.63 |
| white bread | 1.02 | 0.63-1.67 | 0.93 | 1.02 | 0.6-1.71 | 0.96 | 1.47 | 0.72-3.02 | 0.29 |
| Whole grain bread | 1.37 | 0.78-2.41 | 0.27 | 0.98 | 0.57-1.67 | 0.94 | 0.88 | 0.43-1.77 | 0.72 |
| whole milk | 0.95 | 0.54-1.64 | 0.84 | 0.99 | 0.57-1.73 | 0.98 | 1.36 | 0.66-2.78 | 0.41 |
| yoghurt | 0.89 | 0.55-1.44 | 0.62 | 1.23 | 0.75-2.02 | 0.42 | 1.79 | 0.87-3.67 | 0.11 |

*Variable has a significant effect (*p*<0.05)
Note: ordinal regression was used to examine the association between food information sources and dietary intake. Age, sex, education other food information sources, BMI, food allergies, intolerances and restrictions regarding animal products (e.g. vegetarian, flexitarian) were taken in as covariates

Appendix 4c: The relation between food media information sources and dietary intake
(n=1115) [association between media and diet, Belgium, 2022]

|  | Traditional | | |
| --- | --- | --- | --- |
|  | OR | 95% C.I. | *p-value* |
| candies | 1.31 | 0.77-2.21 | 0.32 |
| cereal | 0.65 | 0.37-1.14 | 0.14 |
| cheese | 1.14 | 0.69-1.9 | 0.61 |
| crisps | 1.00 | 0.59-1.68 | 0.99 |
| energy drinks | 1.19 | 0.53-2.71 | 0.67 |
| fish | 0.74 | 0.46-1.2 | 0.23 |
| fries | 1.23 | 0.73-2.06 | 0.44 |
| fruit | 1.31 | 0.76-2.23 | 0.33 |
| legumes | 1.25 | 0.77-2.04 | 0.37 |
| light soda | 0.68 | 0.40-1.15 | 0.15 |
| milk based drinks | 0.78 | 0.39-1.58 | 0.49 |
| non proc. red meat | 0.95 | 0.58-1.58 | 0.86 |
| non proc. white meat | 0.91 | 0.55-1.48 | 0.69 |
| nuts and seeds | 0.91 | 0.56-1.47 | 0.69 |
| pastry | 1.20 | 0.73-1.99 | 0.48 |
| plant based dairy | 0.54 | 0.28-1.04 | 0.07 |
| potatoes | 2.64 | 1.57-4.47 | 0.00 |
| proc. meat replacers | 0.89 | 0.47-1.67 | 0.71 |
| proc. red meat | 1.01 | 0.61-1.68 | 0.97 |
| proc. white meat | 0.73 | 0.45-1.2 | 0.22 |
| skimmed/semi-skimmed milk | 0.82 | 0.5-1.35 | 0.44 |
| soda | 0.90 | 0.53-1.51 | 0.68 |
| unproc. meat replacers | 0.86 | 0.45-1.64 | 0.66 |
| vegetables | 1.59 | 0.87-2.9 | 0.14 |
| water | 2.03 | 0.83-4.98 | 0.12 |
| white bread | 0.73 | 0.43-1.23 | 0.24 |
| Whole grain bread | 1.60 | 0.86-2.97 | 0.14 |
| whole milk | 1.16 | 0.68-1.98 | 0.58 |
| yoghurt | 0.89 | 0.54-1.49 | 0.67 |

*Variable has a significant effect (*p*<0.05)
Note: ordinal regression was used to examine the association between food information sources and dietary intake. Age, sex, education other food information sources, BMI, food allergies, intolerances and restrictions regarding animal products (e.g. vegetarian, flexitarian) were taken in as covariates
